# Life course differences in heavy episodic drinking behaviors across age, gender, and sexual identity in the United States

**DOI:** 10.1016/j.abrep.2023.100495

**Published:** 2023-05-13

**Authors:** Patrick Janulis

**Affiliations:** a Department of Medical Social Sciences, Northwestern University, USA; b Institute for Sexual and Gender Minority Health and Wellbeing, Northwestern University, USA

## Abstract

• This study examines heavy episodic drinking across age, gender, and sexual identity.
• Gay/lesbian and bisexual females experience persistent disparities across age.
• Gay and bisexual males experience age specific disparities.
• Sexual minority adults in the US report complex patterns of heavy alcohol use.
• Heavy drinking disparities do not mirror patterns of alcohol use disorders.

## 1 Introduction

Rates of substance use vary considerably across sex, gender, and sexual identity with elevated rates observed among sexual minorities, particularly among bisexual individuals (Schuler, Rice, Evans-Polce, & Collins, 2018). For alcohol, this includes higher prevalence of drinking (Shokoohi et al., 2022) and greater likelihood of heavy episodic drinking (HED; Schuler, Prince, Breslau, & Collins, 2020) among sexual minorities. Relatedly, rates of alcohol use disorders (AUDs; Peralta et al., 2019, Schuler et al., 2018) and severity of AUDs (Boyd, Veliz, Stephenson, Hughes, & McCabe, 2019) are similarly elevated among sexual minorities. Several studies have found that sexual minority youth are more likely to start drinking at younger ages (Phillips et al., 2019, Schuler and Collins, 2019, Talley et al., 2019) and this may help explain elevated rates of negative alcohol outcomes (e.g., AUDs) given that early age of drinking onset is associated with the development of AUDs (Cheng et al., 2016, Lopez-Quintero et al., 2011). Supporting this contention, several (Coulter et al., 2018, Marshal et al., 2009, Marshal et al., 2012), but not all (Dunbar et al., 2022a, Dunbar et al., 2022b, Talley et al., 2012), cohort studies have identified sharper increases in alcohol use trajectories among sexual minorities in early adulthood.

Accordingly, it is essential to identify life course differences in alcohol use across sexual identity, particularly in representative samples, to best inform approaches to combating higher rates of alcohol use among sexual minorities compared to heterosexual peers (i.e., alcohol use disparities). One recent study (Fish & Exten, 2020) found that sexual minority adults had elevated rates of AUD compared to their heterosexual peers but AUD patterns varied considerably across ages. Sexual minority males and females had the highest prevalence of AUDs in the mid to late 20 s, with females also witnessing a second AUD peak in their 40–50 s (Fish & Exten, 2020). Another study (Peralta et al., 2019) found similarly elevated rates of AUDs among sexual males and females emerging in early adulthood and peaking in the late 20s. These distinct trajectories are likely strongly influenced by the myriad of contextual factors including discrimination (Cerezo & Ramirez, 2021), stigma (Mereish & Miranda, 2019), and stress (Kalb, Roy Gillis, & Goldstein, 2018) that uniquely impact each group at different ages (Peralta et al., 2019).

Yet, AUDs are only one indicator of alcohol use severity and harm. For example, the amount and frequency of drinking helps capture severity of alcohol use for more moderate drinkers (Borges et al., 2010). In addition, the amount of drinking on the heaviest occasions is a strong indicator of risk of developing an AUD (Linden-Carmichael, Russell, & Lanza, 2019). Recognizing this nuance, other indicators of severity, such as number of heavy drinking days, has been increasingly utilized to measure alcohol harm and for evaluating the effectiveness of AUD treatments (Falk et al., 2010, Kiluk et al., 2019). As noted, HED is more common among sexual minorities (Schuler et al., 2020), particularly among bisexual women (Shokoohi et al., 2022). Given that noted differences in AUD disparities across the lifespan (Fish and Exten, 2020, Peralta et al., 2019), the magnitude of HED disparities may also vary by age. However, prior studies (Schuler et al., 2018) have only examined wide age bands likely due to small sample sizes for sexual minority groups. Building on this work, the current study seeks to estimate the prevalence of HED and frequent HED across age, gender, and sexual identity to carefully document life course differences in these behaviors in a nationally representative sample to best inform efforts to understand and reduce these disparities.

## 2 Methods

### 2.1 Study population and sample

The NSDUH is a yearly survey administered by the Substance Abuse and Mental Health Services Administration (SAMHSA). Using a multistage probability sampling design, this survey attempts to represent the civilian non-institutionalized population 12 years and older in all 50 US States and the District of Columbia. While extensive details can be found elsewhere (SAMHSA, 2020), participants complete the survey using audio computer assisted self-interviews (ACASI) and typical annual response rates are ∼70 %. Data for the current analysis comes from 2015 to 2020, as data on sexual identity was first collected in 2015. The current analysis is limited to participants 18 and older because only these participants were asked about their sexual identity.

### 2.2 Measures

#### 2.2.1 Heavy episodic drinking

The number of days of heavy episodic drinking was measured using the following question, “During the past 30 days, that is since [DATE], on how many days did you have [4 or more]/[5 or more] drinks on the same occasion? By “occasion,” we mean at the same time or within a couple of hours of each other,” with 4 drinks being threshold for HED among female participants and 5 drinks being the threshold among male participants. Prevalence of HED was calculated from this variable indicating the presence of 1 or more HED days in the past 30 days while frequent HED was defined as 5 or more HED episodes in the past 30 days (Greene, Johnson, Rosen, German, & Cohen, 2021).

#### 2.2.2 Sexual identity

Sexual identity was measured using the following question, “Which one of the following do you consider yourself to be?” with response options being: “Heterosexual, that is, straight”, “Lesbian or Gay”, and “Bisexual.” Participants who responded, “Don’t Know”, refused to answer, or left the answer blank were excluded from the analysis.

#### 2.2.3 Demographics

Several demographic variables are included in the analysis including gender, age, and race/ethnicity. Gender was designated by the interviewer based on the perceived gender identity of the participant. Age is a restricted variable in the public use data, so the following bins were used: 18–20, 21–25, 26–29, 30–34, 35–49, 50 and over. Race/ethnicity included the following categories: non-Hispanic White, non-Hispanic Black/African American, non-Hispanic Native American / Alaskan Native, non-Hispanic Native Hawaiian or Other Pacific Islander, non-Hispanic Asian, non-Hispanic more than one race, and Hispanic.

### 2.3 Analysis

We utilized a series of generalized linear models to estimate differences in our outcomes across age group, gender, and sexual identity, using a binomial distribution. Using these models, we estimated the predicted probability (Graubard & Korn, 1999) and risk ratio across groups (Bieler, Brown, Williams, & Brogan, 2010), with particular emphasis on differences across sexual identity within gender and age-group. All analysis was completed with the NSDUH provided weights that adjust for over-sampling and post-stratification non-response, including appropriate adjustment for multi-year analysis of the NSDUH, and accounting for survey design. All analysis was completed in R using the *survey* package (Lumley, 2004).

## 3 Results

The final sample of participants 18-years and older that provided their sexual identity was 236,145 participants, including 2,615 gay/lesbian females, 9,537 bisexual females, 2,736 gay males, and 2,754 bisexual males.

**Fig. 1:**
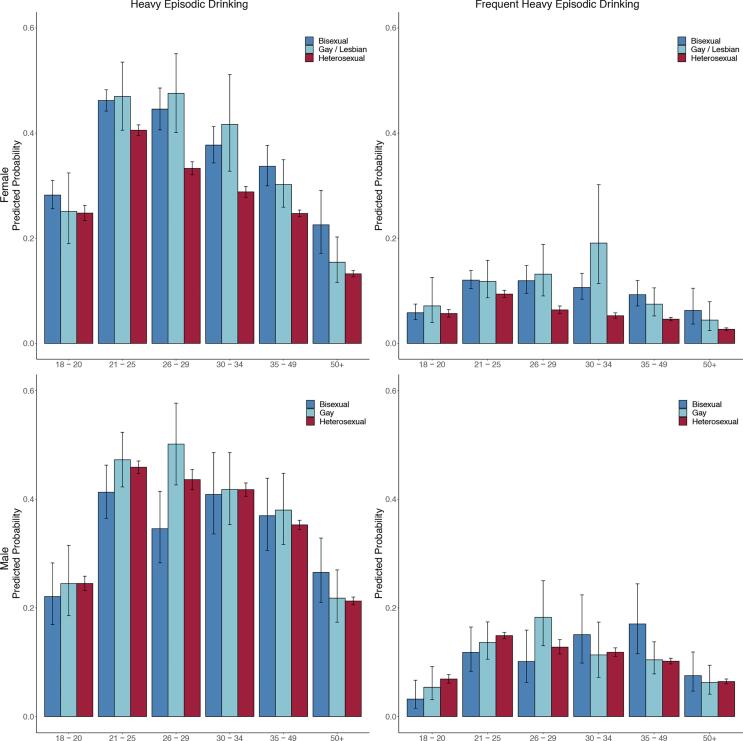
Predicted probability and 95 % CI for heavy episodic drinking (HED) and frequent HED in the past 30 days.

Fig. 1**a** presents the prevalence of HED by sexual identity across age groups for females. Bisexual females report an early disparity of HED (Table 1) at ages 18–20 (IRR = 1.14 [95 % CI: 1.01, 1.28), relative to heterosexual females. However, gay/lesbian females soon surpass bisexual females, although not statistically significantly so, indicating peak prevalence of HED during ages 30–34. Compared to heterosexual females, gay/lesbian females maintain elevated HED prevalence through ages 35–49 (RR = 1.36 [95 % CI: 1.22, 1.52]), while bisexual females maintain elevated prevalence through ages 50 and up (RR = 1.70 [95 % CI: 1.31, 2.22]). For frequent HED (Fig. 1**b**), disparities for bisexual females (RR = 1.28 [95 % CI: 1.08, 1.52]) emerge at ages 21–25 while disparities for gay/lesbian females emerge at ages 26–29 (RR = 2.07 [95 % CI: 1.41, 3.04]). Peak prevalence of frequent HED occurs during ages 21–25 for both bisexual and heterosexual females but bisexual females maintain higher rates of frequent HED throughout all age groups. In contrast, gay/lesbian females indicate the highest levels of frequent HED during ages 30–34, substantially higher than heterosexual females (RR = 3.62 [95 % CI: 2.24, 5.86]), but somewhat rapidly decline with non-significantly different frequent HED by ages 50 and up (RR = 1.64 [95 % CI: 0.91, 2.97]).

**Table 1:** Predicted probability and risk ratio for heavy episodic drinking and frequent heavy episodic drinking.

	HED		Frequent HED	
	Predicted probability [95 % CI]	RR [95 % CI]	Predicted probability [95 % CI]	RR [95 % CI]
**Female**				
18 – 20				
Heterosexual	0.25 [0.23, 0.26]	Ref	0.06 [0.05, 0.06]	Ref
Gay / Lesbian	0.25 [0.19, 0.32]	1.01 [0.78, 1.32]	0.07 [0.04, 0.13]	1.26 [0.71, 2.22]
Bisexual	0.28 [0.26, 0.31]	1.14 [1.01, 1.28]	0.06 [0.05, 0.07]	1.03 [0.77, 1.36]

21 – 25				
Heterosexual	0.41 [0.40, 0.42]	Ref	0.09 [0.09, 0.10]	Ref
Gay / Lesbian	0.47 [0.41, 0.54]	1.16 [1.01, 1.33]	0.12 [0.09, 0.16]	1.26 [0.94, 1.68]
Bisexual	0.46 [0.44, 0.48]	1.14 [1.08, 1.20]	0.12 [0.10, 0.14]	1.28 [1.08, 1.52]

26 – 29				
Heterosexual	0.33 [0.32, 0.35]	Ref	0.06 [0.06, 0.07]	Ref
Gay / Lesbian	0.48 [0.40, 0.55]	1.43 [1.21, 1.68]	0.13 [0.09, 0.19]	2.07 [1.41, 3.04]
Bisexual	0.45 [0.41, 0.49]	1.34 [1.22, 1.47]	0.12 [0.10, 0.15]	1.87 [1.49, 2.35]

30 – 34				
Heterosexual	0.29 [0.28, 0.30]	Ref	0.05 [0.05, 0.06]	Ref
Gay / Lesbian	0.42 [0.33, 0.51]	1.45 [1.16, 1.81]	0.19 [0.11, 0.30]	3.62 [2.24, 5.86]
Bisexual	0.38 [0.34, 0.41]	1.31 [1.22, 1.52]	0.11 [0.08, 0.13]	2.02 [1.57, 2.59]

35 – 49				
Heterosexual	0.25 [0.24, 0.25]	Ref	0.05 [0.04, 0.05]	Ref
Gay / Lesbian	0.30 [0.26, 0.35]	1.22 [1.06, 1.41]	0.07 [0.05, 0.11]	1.62 [1.11, 2.35]
Bisexual	0.34 [0.30, 0.38]	1.36 [1.22, 1.52]	0.09 [0.07, 0.12]	2.01 [1.55, 2.59]

50+				
Heterosexual	0.13 [0.13, 0.14]	Ref	0.03 [0.02, 0.03]	Ref
Gay / Lesbian	0.15 [0.12, 0.20]	1.17 [0.89, 1.53]	0.04 [0.02, 0.08]	1.64 [0.91, 2.97]
Bisexual	0.23 [0.17, 0.29]	1.70 [1.31, 2.22]	0.06 [0.04, 0.10]	2.32 [1.39, 3.88]

**Male**				

18 – 20				
Heterosexual	0.25 [0.23, 0.26]	Ref	0.07 [0.06, 0.08]	Ref
Gay	0.24 [0.17, 0.32]	1.00 [0.77, 1.29]	0.05 [0.03, 0.09]	0.78 [0.46, 1.32]
Bisexual	0.22 [0.17, 0.28]	0.90 [0.70, 1.15]	0.03 [0.02, 0.07]	0.47 [0.23, 0.95]

21 – 25				
Heterosexual	0.46 [0.45, 0.47]	Ref	0.15 [0.14, 0.16]	Ref
Gay	0.47 [0.42, 0.52]	1.03 [0.93, 1.15]	0.14 [0.11, 0.17]	0.91 [0.71, 1.17]
Bisexual	0.41 [0.37, 0.46]	0.90 [0.80, 1.02]	0.12 [0.08, 0.16]	0.79 [0.57, 1.11]

26 – 29				
Heterosexual	0.44 [0.42, 0.46]	Ref	0.13 [0.12, 0.14]	Ref
Gay	0.50 [0.43, 0.58]	1.15 [0.98, 1.34]	0.18 [0.13, 0.25]	1.43 [1.03, 1.98]
Bisexual	0.35 [0.28, 0.41]	0.79 [0.66, 0.95]	0.10 [0.06, 0.16]	0.79 [0.51, 1.23]

30 – 34				
Heterosexual	0.42 [0.41, 0.43]	Ref	0.12 [0.11, 0.13]	Ref
Gay	0.42 [0.35, 0.49]	1.00 [0.85, 1.17]	0.11 [0.07, 0.17]	0.96 [0.61, 1.50]
Bisexual	0.41 [0.34, 0.49]	0.98 [0.88, 1.25]	0.15 [0.10, 0.22]	1.27 [0.85, 1.91]

35 – 49				
Heterosexual	0.35 [0.34, 0.36]	Ref	0.10 [0.10, 0.11]	Ref
Gay	0.38 [0.32, 0.45]	1.08 [0.91, 1.27]	0.10 [0.08, 0.14]	1.02 [0.77, 1.36]
Bisexual	0.37 [0.31, 0.44]	1.05 [0.88, 1.25]	0.17 [0.12, 0.24]	1.67 [1.16, 2.42]

50+				
Heterosexual	0.21 [0.21, 0.22]	Ref	0.06 [0.06, 0.07]	Ref
Gay	0.22 [0.17, 0.27]	1.02 [0.82, 1.27]	0.06 [0.04, 0.09]	0.97 [0.65, 1.46]
Bisexual	0.27 [0.21, 0.33]	1.25 [1.00, 1.56]	0.08 [0.05, 0.12]	1.17 [0.74, 1.85]

Differing patterns emerge for sexual minority men (Fig. 1**c**). Sexual minority males have remarkably similar prevalence of HED for almost all age groups with prevalence peaking around ages 21–29 across all groups, except for bisexual males reporting lower prevalence of HED (RR = 0.79 [95 % CI: 0.66, 0.95]) during ages 26–29 relative to heterosexual males. For frequent HED, bisexual males report lower prevalence during ages 18–20 (RR = 0.47 [95 % CI: 0.23, 0.95]) but mostly increasing prevalence of frequent HED until ages 35–49 where they report significantly higher prevalence (RR = 1.67 [95 % CI: 1.16, 2.42]) relative to heterosexual males. However, gay males report similar rates to heterosexual males except during ages 26–29 when they report the highest prevalence, which is significantly higher (RR = 1.43 [95 % CI: 1.03, 1.98] than heterosexual males.

## 4 Discussion

This study examined the prevalence of HED across age, gender, and sexual identity in the United States. As expected, both sexual minority females and males reported higher levels of HED in some age groups. For females, consistent disparities emerged during the early to late 20 s and are relatively consistently maintained across age groups, with bisexual females slightly but non significantly higher in later ages. However, few disparities existed in sexual minority males compared to heterosexual peers. In fact, bisexual males had some indication of lower prevalence of HED and frequent HED in earlier ages, although higher prevalence of HED in ages 35–49.

These findings provide additional nuance to prior studies examining life course differences in alcohol use behavior among sexual minority adults. For example, one study using 2015 and 2016 NSDUH data found that HED was primarily elevated in bisexual females but was only elevated in young (i.e., 18–24) gay/lesbian females (Schuler et al., 2018). This contrasts with the current analysis that found consistently elevated prevalence of HED and frequent HED among both bisexual and gay/lesbian females. This difference is likely due to the larger sample and more precise age bins used in the current analysis which provides greater granularity to these estimates that vary across age groups.

Interestingly, the patterns of HED observed in this study also do not directly align with recent studies examining patterns of AUD across age, gender, and sexual identity (Fish and Exten, 2020, Peralta et al., 2019). For example, while HED prevalence largely mirror prevalence of AUD peaking in mid to late 20 s, we found frequent HED for both gay/lesbian females and bisexual males stays heightened through years 30–49, diverging from observed prevalence of AUD in these studies. In addition, the lack of disparities in HED and limited disparities in frequent HED among sexual minority males is striking given the substantial and persistent disparities observed in AUDs across these groups (Fish and Exten, 2020, Peralta et al., 2019). This may suggest that contextual factors that impact sexual minority men, such as stigma and discrimination, may heighten the risk for developing an AUD despite reporting minimal differences in levels of drinking intensity. These findings suggest intervention targeting individuals at risk for harmful alcohol use will require careful attention to variation across sexual identity and the specific outcomes of interest (e.g., AUD versus HED), as these behaviors may not reflect a uniform pattern across the lifespan.

Main limitations of the current analysis include that it cannot easily distinguish age, period, and cohort effects given the relatively small number of years with data on sexual identity. Accordingly, age differences could reflect any combination of these effects and additional data are required to further interrogate the observed differences. Relatedly, the age bins available in the public dataset also limit the granularity of analysis across age. Finally, the NSDUH provides a single binary variable to indicate the participant’s gender as perceived by the interviewer with no information about gender minority individuals (e.g., transgender individuals). This limits our ability to explore gender differences.

Despite these limitations, this study adds to the accumulating evidence (Fish and Exten, 2020, Peralta et al., 2019) suggesting individuals with sexual minority identities experience distinct life course patterns of alcohol use. Future studies should continue to explore these differences with particular attention to sustained disparities among sexual minority females and, to a lesser extent, sexual minority males in mid adulthood.

## Funding

This work was supported by grants from the National Institute on Drug Abuse (R01DA055502) at the National Institutes of Health.

## CRediT authorship contribution statement

**Patrick Janulis:** Conceptualization, Formal analysis, Writing – original draft, Funding acquisition.

## Declaration of Competing Interest

The authors declare the following financial interests/personal relationships which may be considered as potential competing interests: [Patrick Janulis reports financial support was provided by National Institute on Drug Abuse.].

## Data Availability

Data are publicly available
